# A spatio-temporal land use and land cover reconstruction for India from 1960–2010

**DOI:** 10.1038/sdata.2018.159

**Published:** 2018-08-14

**Authors:** Simon Moulds, Wouter Buytaert, Ana Mijic

**Affiliations:** 1 Centre for Water Systems, College of Engineering, Mathematics and Physical Sciences, University of Exeter, Exeter, EX4 4QF, UK; 2 Department of Civil and Environmental Engineering, Imperial College London, London, SW7 2AZ, UK

**Keywords:** Environmental chemistry, Geography, Hydrology

## Abstract

In recent decades India has undergone substantial land use/land cover change as a result of population growth and economic development. Historical land use/land cover maps are necessary to quantify the impact of change at global and regional scales, improve predictions about the quantity and location of future change and support planning decisions. Here, a regional land use change model driven by district-level inventory data is used to generate an annual time series of high-resolution gridded land use/land cover maps for the Indian subcontinent between 1960–2010. The allocation procedure is based on statistical analysis of the relationship between contemporary land use/land cover and various spatially explicit covariates. A comparison of the simulated map for 1985 against remotely-sensed land use/land cover maps for 1985 and 2005 reveals considerable discrepancy between the simulated and remote sensing maps, much of which arises due to differences in the amount of land use/land cover change between the inventory data and the remote sensing maps.

## Background & Summary

India’s population has risen dramatically in recent decades, from 361 million in 1951 to 1,221 million at the last national census in 2011^1^), driving substantial environmental change^2^. Increasing population density and a changing economy have resulted in urban development and expansion^3^, while India’s green revolution, initiated in the mid-1960s to achieve food security for its growing population, has resulted in the transformation of natural vegetation and rainfed agriculture to intensively managed agricultural systems^4,5^. Land use/land cover change (henceforth “land change”) has important consequences for biodiversity and the sustainability of ecosystem services upon which individuals and communities depend^6^. Cumulatively, it is a major driver of global and regional environmental change^7^, influencing the surface energy and water balance^8,9^ as well as global biogeochemical cycles^10^. Since the rate and magnitude of land change varies over space and time^2^, spatially explicit, historical datasets are necessary to accurately quantify the various environmental and societal impacts of change^11–13^, support planning decisions and inform predictions about the quantity and location of future change.

Remote sensing provides one data source for mapping land change^14^. Recently Roy *et al.*^15^ developed land use/land cover maps of India for 1985, 1995 and 2005 using multitemporal satellite data from various instruments. Developing such a dataset is resource intensive and severely constrained by the quality and availability of satellite data and ground truth points that are valid for the time period considered^15^. Moreover, the launch of the first Landsat instrument in 1972 represents the earliest time at which remote sensing could be used for land use/land cover mapping. As a result of these factors, existing work to document historical land use/land cover at global and regional scales have used various algorithms to combine contemporary land use/land cover maps from remote sensing with national and sub-national inventory data^13^. Global datasets include the HYDE database^16^ and the Earthstat global cropland and pasture dataset^17^. These products have a 5 arcminutes×5 arcminutes resolution (approximately 10 km at the Equator) and cover the period 10,000BC-2005AD and 1700-2007, respectively. In India, global datasets such as these rely on land use/land cover inventory data aggregated to state and national levels because of the difficulties in obtaining district-level data collected before 1998. As a result, they do not adequately capture the variability in the rate of change between districts, which can be considerable. For example, while the northern State of Uttar Pradesh shows a net increase in the area of agricultural land between 1956 and 2010, the area in 20% of district units (*n*=8) has decreased.

Addressing this deficiency,^2^ developed an historical dataset for India which combines a contemporary land use/land cover map based on Resourcesat-1 satellite imagery with a commercially available dataset of district-level inventory data. The dataset has a spatial resolution of 5 arcminutes × 5 arcminutes and covers the period 1880–2010. Central to the approach adopted in this case as well as the global datasets mentioned previously is the assumption that the spatial distribution of historical land use/land cover is the same in relative terms as the present distribution^2^. However, this does not adequately reproduce historical change because it fails to account for changes in the relative importance of the various factors influencing land change over time^13^. Spatially explicit land change models, which simulate future or historical land change based on statistical analysis of the quantitative relationship between the contemporary land use/land cover distribution and various socioeconomic and biophysical covariates^18^, have been suggested as an essential next step to improve historical land use/land cover reconstructions^2,13^.

This paper presents a new dataset showing historical land change in India between 1960–2010. It uses the HIstoric Land Dynamics Assessment (HILDA) land change model^19,20^ to combine district-level inventory data with a state of the art high-resolution land use/land cover product for the year 2005^15^. The resulting dataset, which has a spatial resolution of 100 m 100 m and provides a land use/land cover map for each year of the study period, shows the evolution of cropland, forest, grassland, shrubland, wasteland, barren land and urban areas. It is the first publicly available, spatially explicit dataset derived from district-level inventory data showing land change in India during the second half the twentieth century.

## Methods

### The HILDA land change model

The HIstoric Land Dynamics Assessment (HILDA) land change model^19,20^ simulates land change over a spatial grid in which individual pixels belong to a single land use/land cover type. HILDA was originally developed for reconstructing historical land change in Europe^19^. While the model is relatively simple compared to more sophisticated approaches^21,22^, it is well suited to modelling land change over large study regions because it is computationally efficient and guaranteed to produce a solution which satisfies the regional land change requirement. The allocation procedure is based on statistical analysis of the quantitative relationship between the contemporary distribution of land use/land cover and various socioeconomic and biophysical covariates. The resulting regression models are used to estimate the probability of pixels belonging to the respective land use/land cover type given the underlying biophysical and socioeconomic conditions. At each time point in the simulation period the model spatially allocates a given quantity of change amongst the various land use/land cover types. Allocation proceeds in a hierarchical way according to the perceived relative socioeconomic value of the various land use/land cover types under consideration. Land uses with increasing area at the regional level are only allowed to expand to areas currently occupied by land uses with lower socioeconomic value. In this case, *n* pixels with the highest probability of belonging to the land use, but which do not currently belong to to it, are selected to change. In the case of land uses with decreasing area, *n* pixels with the lowest probability of belonging to the land use are nominated for conversion to an alternative land use.

### Input data

#### Administrative boundaries

Contemporary district and state boundaries for India were obtained from version 2.7 of the Global Administrative Areas Database (GADM; http://www.gadm.org/). In order to account for boundary changes that occurred during the study period, contemporary GADM boundaries were manually adjusted until they corresponded with boundaries that were consistent over the course of the study period. Locations of boundary changes were identified from a scanned, historical geological map from the European digital archive on soil maps (EuDASM)^23^ showing district boundaries in 1971.

#### Land use and land cover inventory data

The amount of land change in each district and at each time point during the study period was derived from district-level land use/land cover inventory data. In India, data about land use/land cover are collected at the village level on an annual basis and aggregated to district and state administrative levels. The compilation of the village level data is coordinated by the Directorate of Economics and Statistics of India’s Ministry of Agriculture. Data for the entire study period, except the period 1993–1997 which for unknown reasons was not available, was purchased from Indiastat (https://www.indiastat.com/), a commercial organisation which distributes socio-economic data about India. An additional source of district-level survey data for 19 States and Union Territories for the period 1966 to 2009 was obtained from the Icrisat Village Dynamics in South Asia (VDSA) project (http://vdsa.icrisat.ac.in/). This data originates from the same ultimate source as the Indiastat data but, as part of the VDSA project, has already undergone some data quality assurance and, moreover, includes data for the period 1993–1997.

#### Observed land use/land cover data

Observed land use/land cover information was obtained from Roy *et al*.^15^; a dataset providing maps at 100 m×100 m resolution for 1985, 1995 and 2005 derived from multi-temporal satellite data from Landsat, IRS 1C-LISS III and Resourcesat1 instruments. According to the accuracy assessment conducted by Roy *et al*.^15^, which utilised 12,606 ground truth points, the 2005 map had an overall mapping accuracy of 94.46% and a Kappa coefficient of 0.9445. While the accuracy of the 1985 and 1995 map was not quantified due to the lack of ground truth data for these time points^15^, argue that the accuracy is likely to be similar to that of the 2005 map.

#### Biophysical and socio-economic predictor variables

The HILDA model is based on statistical relationships between land use/land cover and spatially-explicit biophysical and socio-economic predictor variables. In total, 16 covariables were used (Table 1). Data on topography (elevation, slope, aspect), which is known as an important determinant of land use/land cover, was derived from the HydroSHEDS database, which itself is derived from the NASA Shuttle Radar Topographic Mission digital elevation model. Bioclimatic variables assumed to influence the spatial configuration of the landscape were obtained from the Bioclim dataset^24^.

A map of population density for 2010 at 100 m resolution was obtained from the WorldPop database^25^. Accessibility was primarily represented by a map showing the estimated travel time to the nearest city with a population of 50,000 or more for the year 2000^26^. Additionally, distance to roads was estimated using a map of major roads from the Global Roads Open Access Dataset^27^. These predictor variables were treated as static in time due to a lack of corresponding datasets for historical time points.

### Data preparation

The geological map showing district boundaries for 1971 was geolocated and used together with historical records to identify changes in administrative boundaries over the course of the study period. Contemporary district polygons from GADM were manually dissolved until the resulting area corresponded with boundaries shown by the historical map. As a result of the changes the mean district unit area increased from 5307 km^2^ (*n*=594, *σ*=4673 km^2^) in the original GADM map to 10650 km^2^ (*n*=296, *σ*=8777 km^2^) in the modified version.

Land use/land cover maps for India (1985, 1995, 2005) were reprojected from Universal Transverse Mercator to Lambert Conformal Conic projection using nearest neighbour interpolation. The resulting images were reclassified to nine land use/land cover types (Table 2). Biophysical and socioeconomic covariates were resampled to the same projection and spatial resolution as the fractional land use/land cover maps using bilinear interpolation. Lastly, the modified administrative area map was reprojected to Lambert Conformal Conic projection and each polygon was converted to a raster image with the same spatial resolution as the other spatial data.

Inventory data from Icrisat and Indiastat were homogenized by checking for inconsistencies, identifying different spellings of administrative unit names, and applying consistent formatting. Although the inventory data for several districts was incomplete, either because of boundary changes or missing data, all district inventory data were mapped to a common annual time series between 1960–2010. The resulting data files were aggregated to correspond with the district units of the modified GADM administrative area map. This resulted in two new data files in which district units were associated with a unique time series of land use/land cover data from Indiastat and Icrisat respectively.

The inventory data contains information about nine land use/land cover classes: (a) forests, (b) area under non-agricultural uses, (c) barren and unculturable land, (d) permanent pastures and other grazing land, (e) miscellaneous tree crops, (f) culturable waste land, (g) fallow land other than current fallows, (h) current fallows, and (i) net area sown. This classification scheme was simplified to provide the demand scenario input to the land change model (Table 2). The change in urban area was estimated by multiplying the time series of area under non-agricultural uses, which includes artificial surfaces, by a scale factor equal to the urban area from the 2005 observed map divided by the area under non-agricultural uses for 2005 (ref. 2). The area of water and snow and ice are also obtained from the 2005 observed map and, assuming they remain constant during the study period, subtracted from the area under non-agricultural uses. Any remaining area under non-agricultural uses was assigned to barren land.

Data quality control was performed at the district level by plotting the time series of each land use/land cover class, identifying quality issues and infilling or correcting problems as appropriate. Various types of error were identified, including: (1) data points labelled as one category but in fact belonging to another; (2) data points labelled with the wrong area units; (3) missing data points and outliers. Misclassified data points were reclassified to the correct category while data points labelled with the wrong units were multiplied by an appropriate conversion factor. Missing data points and outliers were infilled using linear interpolation or, if the problem occurred in the first or last data points, last observation carried forwards.

The final processing step was to calibrate the district-level time series data with land use/land cover quantities from the 2005 land use/land cover map, following the approach of Tian *et al*.^2^. This was necessary because the spatial allocation procedure requires the total area of each land cover type at the initial time point to be the same in the initial condition map (i.e. the 2005 map from Roy *et al*.^15^) and the non-spatial demand scenario (i.e. the time series dataset derived from the inventory data). Discrepancies between the respective values may arise for a number of reasons, including inaccurate reporting of certain land cover types in the inventory data or misclassification of pixels in the remote sensing dataset. In each district unit a calibration factor was determined for each land use category by dividing the quantity from the 2005 land use/land cover map with the corresponding quantity from the inventory data. The time series of each category was then multiplied by the calibration factor to ensure the area of each land cover type between the 2005 land cover map and the inventory data in each district corresponded. Differences between the district area and the sum of the various land use/land cover types at each time point were resolved by visually identifying the land uses responsible for the discrepancy and adjusting the respective areas accordingly.

### Land change modelling procedure

**Figure 1:**
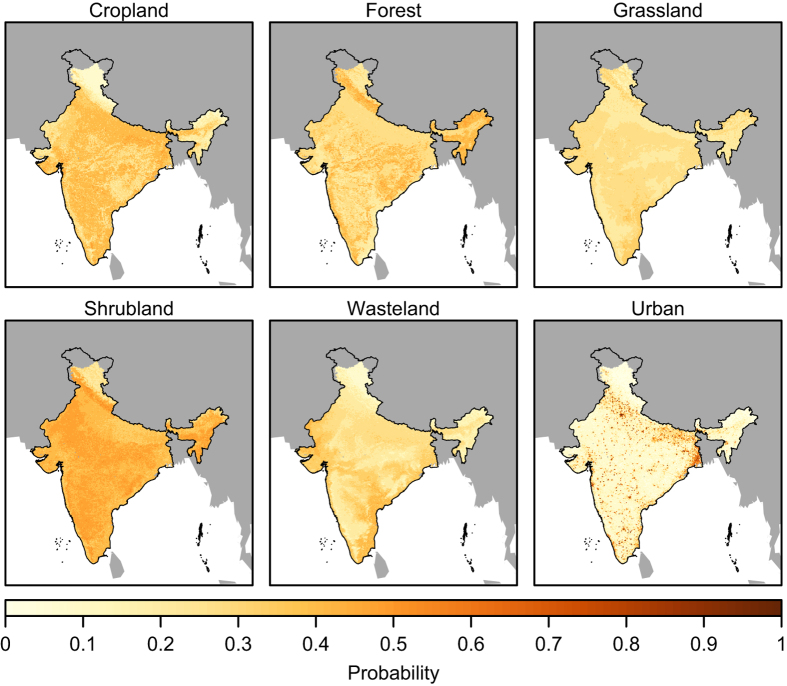
Probability surfaces for cropland, forest, grassland, shrubland, wasteland and urban, calculated with random forest models. Barren land does not require a probability surface because it is allocated last, while water and snow and ice are considered constant during the simulation.

Random forest, a non-parametric ensemble learning method that is robust against overfitting and efficiently handles datasets with a large number of input variables^28,29^, was used to fit models of the spatial distribution of land use/land cover in 2005 with the biophysical and socio-economic predictor variables. The random forest algorithm constructs multiple classification trees using different bootstrap samples of the data^28^, splitting tree nodes using the best split among a random selection of variables^28,30^. For regression tasks the results from individual trees are combined by taking the mean prediction. The statistical analysis was performed on a stratified random subset containing 1000 pixels from each land use/land cover class. This subset was further divided into training and test sets according to a 70:30 ratio. All models (cropland, forest, grassland, shrubland, wasteland, urban) were trained and tested on the same data partitions. A statistical model was not required for barren land because, as the land use/land cover class with the lowest socioeconomic value, it is automatically assigned to unallocated pixels once the other classes have been modelled. Fig. 1 shows the suitability surfaces for each land use/land cover class.

**Figure 2:**
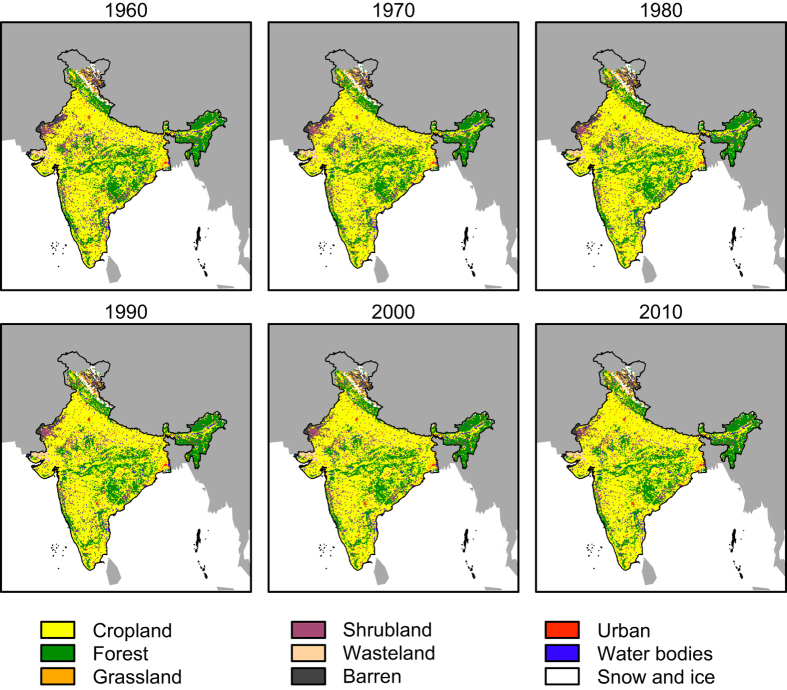
Simulated land use/land cover maps at decadal time steps over the study period.

The HILDA model was applied to each district using demand scenarios constructed from the inventory data. This approach means that variability in the rate of land change between districts is implicitly taken into account, while the annual temporal resolution of the inventory data ensures that the temporal dynamics of land change in each district is properly represented. Seven land use/land cover types were modelled in the following order: urban land, cropland, grassland, shrubland, wasteland and barren land. Water and snow and ice classes were included in the simulation but assumed to be constant over the study period. The resulting district-level land use/land cover maps were combined to generate an historical land change dataset for the entire Indian subcontinent. Fig. 2 shows the land use/land cover maps at decadal time steps between 1960 and 2010.

### Code availability

All code used in the present study is provided under the GNU General Public Licence alongside the resulting datasets (see Data Records section). Spatial input data was processed in GRASS GIS. The R package *randomForest*, which provides an interface to the original Fortran code^28^, was used for statistical analysis. Land change modelling was carried out using the lulcc software package^31^. The implementation of HILDA is adapted from the original model description^19^. The version of *lulcc* used in the present study is provided in the data repository; in addition, a development version resides on the first author’s GitHub account (https://github.com/simonmoulds/r_lulcc2).

## Data Records

The dataset provides annual maps of each land use/land cover type for the period 1960-2010 in Lambert Conformal Conic projection (Data Citation 1). Land use/land cover inventory data from Indiastat and ICRISAT have been converted to a consistent format and placed in an R data package which is included alongside the spatial data. The archive also contains the scripts and input data (or instructions about how to obtain the data) together with instructions for reproducing the dataset.

## Technical Validation

### Statistical analysis

**Figure 3:**
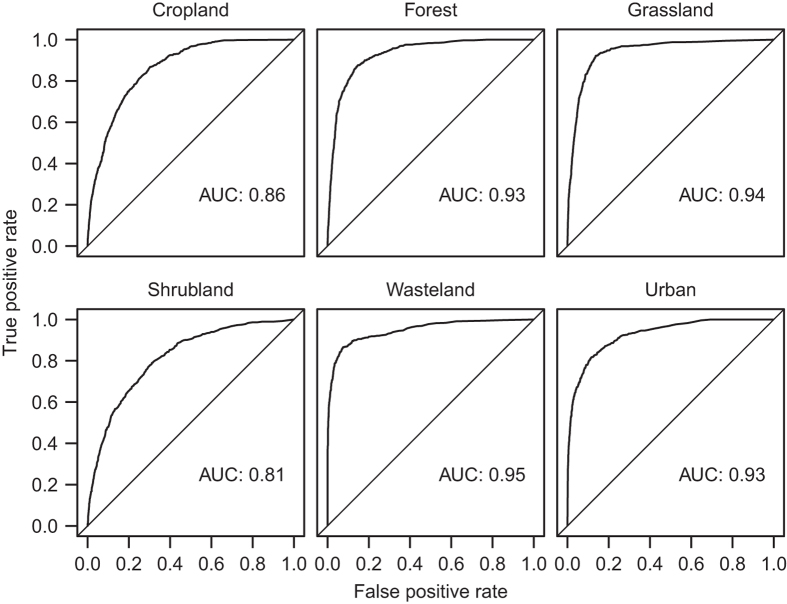
Receiver operator characteristic curves and associated area under the curve (AUC) for the random forest models of cropland, forest, grassland, shrubland, wasteland and urban land.

The statistical models were evaluated using the receiver operator characteristic (ROC) curve; a graphical technique which is commonly used to measure the performance of presence-absence models^32^. The curve is constructed by plotting the true positive rate (sensitivity) against the false positive rate (1−specificity) for various cut-off points. It is summarised by estimating the area under the curve (AUC) value, where an area of 1 indicates a perfect model and a value of 0.5 indicates a random model. The ROC curves for the six random forest models are shown in Fig. 3, demonstrating that the models simulate the occurrence of the respective land uses with an acceptable degree of accuracy.

### Land use change modelling

**Figure 4:**
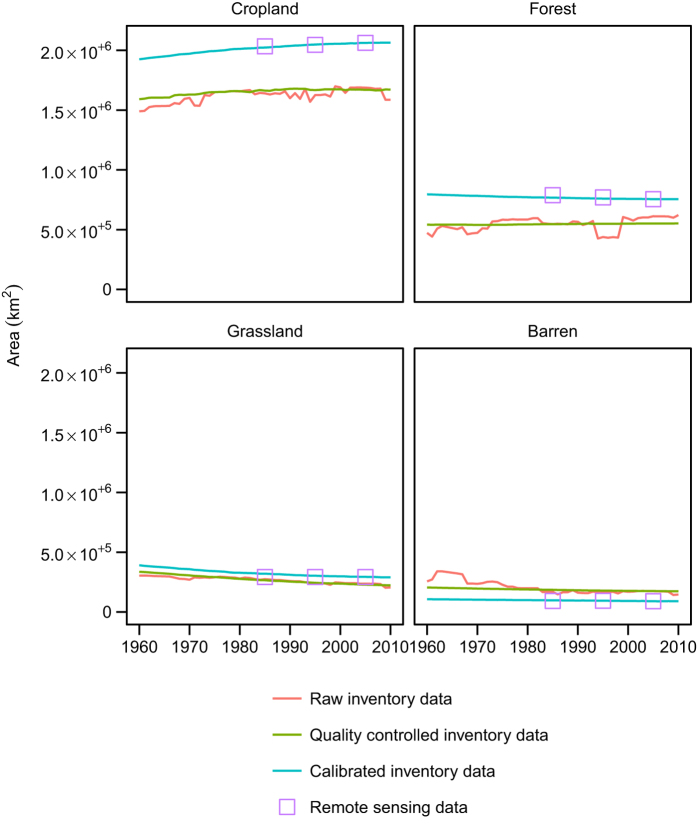
Comparison of the area of cropland, forest, grassland (including shrubland and wasteland) and barren land at the national level considering the raw inventory data and the inventory data after quality control and calibration, as well as the corresponding areas from the remote sensing product.

**Figure 5:**
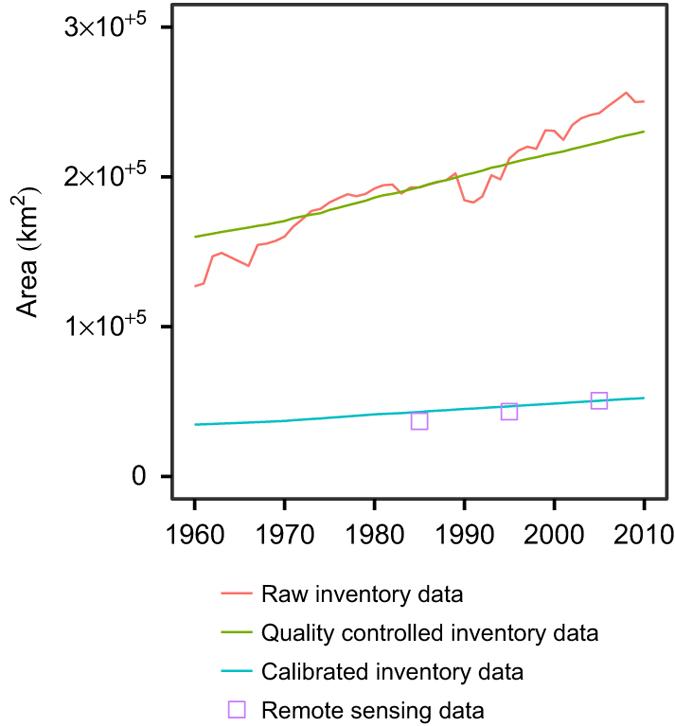
Comparison of the area of urban areas at the national level considering the inventory data in raw, quality controlled and calibrated forms, as well as the urban area from the remote sensing product. The land use/land cover class corresponding to urban area also includes various non-urban, non-agricultural land uses.

Figures 4 and 5 compare the aggregated demand scenarios of the various land use/land cover types with the raw inventory data and the corresponding areas from the 1985, 1995 and 2005 remote sensing maps. These plots demonstrate the discrepancy between land use/land cover areas according to remote sensing on the one hand and inventory data on the other. In addition, they show that while the trajectory of cropland, grassland and barren land observable in the raw data is maintained in the calibrated demand scenarios, the direction of change of forest is different in the two time series. The rate of change of grassland (which in this plot includes shrubland and wasteland) between 1985 and 2005 is more gradual in the remote sensing data compared to the inventory data, which may arise because of uncertainty about the transition between shrubland and forest.

**Figure 6:**
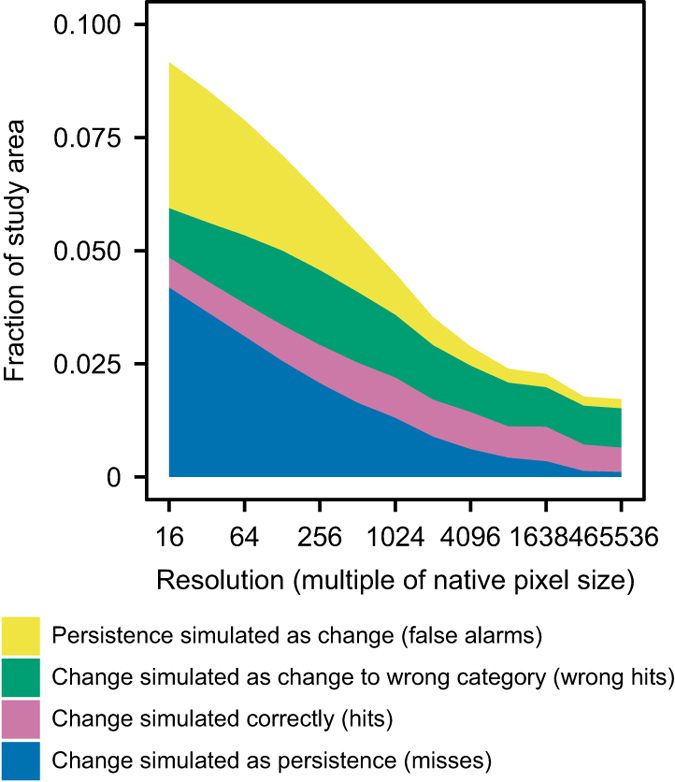
Components of agreement and disagreement at multiple resolutions, excluding persistence simulated correctly.

The spatial distribution of land change was validated for 1985; the earliest time point for which a reference land cover map was available. The validation was performed using the method developed by Pontius *et al*.^33^, in which a simulated land cover map for time 2 is compared with reference maps for time 2 and time 1. In the present study, the simulated map for 1985 was compared with reference maps for 1985 and 2005, which were taken from the remote sensing dataset described previously^15^. The comparison was performed at multiple resolutions in order to distinguish between minor allocation disagreement (disagreement at native resolution which is counted as agreement at a coarser resolution) with major allocation disagreement (disagreement at the native and coarse resolution). Figure 6 shows the components of agreement and disagreement between the three maps at multiple resolutions, while Table 3 shows the value of each component of agreement and disagreement at the various spatial resolutions. The comparison was not carried out on resolutions finer than 16 times the native resolution because of difficulties performing the analysis on very large raster maps. At 16 times the native resolution there is 91% agreement between the observed and simulated maps for 1985, with the vast majority of agreement arising from correctly simulated persistence (i.e. pixels which have not changed between 1985 and 1995). At this resolution the largest source of disagreement between the observed and simulated maps is change simulated as persistence. Of all pixels that were observed to change between 1985 and 2005, the simulated map for 1985 is in agreement with the reference map in around 6% of cases at 16 times the native resolution, rising to 19% at 1,024 times the native resolution (i.e. around 100 km×100 km resolution) and 35% at the coarsest resolution. Overall disagreement is 9% at the finest resolution considered, 3.8% at 1,024 times the native resolution and 1.1% at the coarsest resolution. At 65,536 times the native resolution the entire study area is represented as one pixel, which necessarily means that disagreement is entirely due to quantity disagreement; in other words, disagreement between the inventory data and the remote sensing data about the total quantity of change when it is aggregated nationally. Since quantity disagreement is consistent across all resolutions, around 10% of the disagreement at 16 times the native resolution is the result of quantity, rather than allocation, disagreement.

## Usage Notes

It is envisaged that the dataset will be used in a variety of contexts across disciplines. For example, it may be used in combination with impact assessment models to quantify the environmental impacts of land use change. Such analyses may inform policies designed to support sustainable agricultural development, urban expansion and the protection of ecosystem services. Historical land cover maps are an essential input to assessments of global and regional change because land cover influences the water and energy fluxes. Moreover, land use/land cover maps are a necessary first step towards developing datasets of land management and land use intensity^10^. Thus, a valuable extension to the present dataset would be the inclusion of more information about specific land management practices such as irrigation and multiple cropping.

The dataset is associated with a large amount of uncertainty from various sources. In particular, the raw district-level inventory data contains a large amount of noise which had to be manually removed before supplying it to the land change model. To ensure decisions made during quality control are open, reproducible and amendable the data processing scripts are included alongside the land use/land cover maps. The HILDA land change model is strongly influenced by the statistical analysis of the spatial distribution of contemporary land use/land cover. The random forest models employed in the present study use several publicly available datasets (Table 1). Recognising that other members of the community may have access to alternative, or additional, datasets outside the public domain, the source code includes clear instructions about incorporating additional data into the statistical analysis. Users of the dataset should be aware that in the present analysis the explanatory factors of population density, accessibility and distance to roads have been treated as static in time due to the lack of historical, spatially explicit data showing the evolution of these variables during the study period.

Individual land use/land cover maps are provided as GeoTIFF files which can be processed in standard Geographical Information System (GIS) software as well as data processing languages such as R and Python. To assist users who may require maps at relatively coarse resolutions (e.g. for supplying to Earth system models), the data repository includes maps in unprojected format at 5 arcminutes × 5 arcminutes spatial resolution, showing the fraction of each pixel belonging to the various land use/land cover types. Users should be aware that reprojecting the maps from the original Lambert Conformal Conic projection will change the aggregate area of each land use/land cover type.

## Additional information

**How to cite this article**: Moulds, S. *et al*. A spatio-temporal land use and land cover reconstruction for India from 1960–2010. *Sci. Data* 5:180159 doi: 10.1038/sdata.2018.159 (2018).

**Publisher’s note**: Springer Nature remains neutral with regard to jurisdictional claims in published maps and institutional affiliations.

## Supplementary Material



## Figures and Tables

**Table 1:** Biophysical and socioeconomic covariates.

**Variable**	**Source**	**Spatial resolution**	**Time**
Topography	HydroSHEDS	3 arcseconds	N/A
Population density	WorldPop^25^	30 arcseconds	2005
Road network	gROADS^27^	N/A	Various
Travel time	[26]	30 arcseconds	2000
Annual mean temperature	Bioclim	30 arcseconds	1970–2000
Mean diurnal range	Bioclim	30 arcseconds	1970–2000
Isothermality	Bioclim	30 arcseconds	1970–2000
Temperature seasonality	Bioclim	30 arcseconds	1970–2000
Maximum temperature of warmest month	Bioclim	30 arcseconds	1970–2000
Minimum temperature of coldest month	Bioclim	30 arcseconds	1970–2000
Temperature annual range	Bioclim	30 arcseconds	1970–2000
Mean temperature of wettest quarter	Bioclim	30 arcseconds	1970–2000
Mean temperature of driest quarter	Bioclim	30 arcseconds	1970–2000
Mean temperature of warmest quarter	Bioclim	30 arcseconds	1970–2000
Mean temperature of coldest quarter	Bioclim	30 arcseconds	1970–2000
Annual precipitation	Bioclim	30 arcseconds	1970–2000

**Table 2:** Translation of land use/land cover classification schemes of inventory data and remote sensing product to the classes used in the land change modelling procedure.

**HILDA modeling**	**Inventory data**	**Remote sensing data**^15^
Cropland	Net area sown Miscellaneous tree crops Current fallows Fallow land other than current fallows	Cropland Plantations Fallow land
Forest	Forest	Deciduous broadleaf forest Deciduous needleleaf forest Evergreen broadleaf forest Evergreen needleleaf forest Mixed forest Mangrove forest
Grassland	Permanent pasture	Grassland
Shrubland	Culturable wasteland	Shrubland
Wasteland		Wasteland
Barren land	Barren and unculturable land	Barren land Salt pan
Urban	Area under non-agricultural uses	Artificial surfaces
Water bodies	Area under non-agricultural uses	Water bodies Wetlands Aquaculture
Snow and ice	Barren and unculturable land	Snow and ice

**Table 3:** Components of agreement and disagreement at multiple resolutions (fraction of study area).

**Multiple of native resolution**	**(1)**	**(2)**	**(3)**	**(4)**	**(5)**
16	0.042	0.006	0.011	0.037	0.904
32	0.037	0.006	0.013	0.034	0.910
64	0.031	0.007	0.016	0.030	0.917
128	0.026	0.008	0.017	0.025	0.925
256	0.021	0.008	0.018	0.020	0.934
512	0.016	0.009	0.017	0.014	0.944
1024	0.013	0.009	0.015	0.010	0.954
2048	0.009	0.008	0.013	0.007	0.963
4096	0.006	0.008	0.011	0.005	0.971
8192	0.004	0.007	0.010	0.004	0.975
16384	0.003	0.008	0.008	0.003	0.977
32768	0.001	0.007	0.008	0.002	0.982
65536	0.001	0.006	0.008	0.002	0.983
(1) Change simulated as persistence (misses)					
(2) Change simulated correctly (hits)					
(3) Change simulated as change to wrong category (wrong hits)					
(4) Persistence simulated as change (false alarms)					
(5) Persistence simulated correctly (correct rejections).					
